# Chemical Composition and Biological Activities of Methanolic Extract of *Scrophularia Oxysepala* Boiss

**Published:** 2017

**Authors:** Ardalan Pasdaran, Abbas Delazar, Seyed Abdulmajid Ayatollahi, Arsalan Pasdaran

**Affiliations:** a *Medical Plant Processing Research Center, Shiraz University of Medical Sciences, Shiraz, Iran.*; b *Department of Pharmacognosy, School of Pharmacy, Research and Development Center of Plants and Medicinal Chemistry, Guilan University of Medical Sciences, Rasht, Iran****. ***; c *Department of Pharmacognosy, Faculty of Pharmacy, Tabriz University of Medical Sciences, Tabriz, Iran. *; d *Phytochemistry Research Center, Shahid Beheshti University of Medical Sciences, Tehran, Iran. *; e *Department of Pharmacognosy, School of Pharmacy, Shahid Beheshti University of Medical Sciences, Tehran, Iran.*; f *Faculty of Basic sciences, Central Tehran Branch, Islamic Azad University, Tehran, Iran.*

**Keywords:** *Scrophulariaoxysepala*, phenyl ethanol amine, Iridoids glycosides, radical scavenging, general toxicity, antimalarial activity, insecticidal activity

## Abstract

Isolated five known iridoids glycosides (Scrophuloside A, Harpagoside B, 5-hydroxyloganin, 8-O-acetylharpagide and 6-O-methyl,1-glucopyranosyl catalpol), one phenyl ethanoid glycoside (Verbascoside) and a phenyl ethanol amine (2-(4-Chlorobenzyl amino) ethanol) compound from the methanolic extract of aerial parts of *Scrophularia oxysepala* using by high performance liquid chromatographyare based on isocratic and liner gradients by C18 column. The structure elucidations of the isolated compounds were performed by spectroscopic methods including1H-NMR, 13C-NMR, 2 D NMR technique such as HMBC( in deuterated methanol as solvent), GC-MS and UV, also methanolic extract and fractions( fractionated on solid phase extraction on C18 cartridge(Spack-C18)) of this plant was tested for free radical scavenging properties toward the 1, 1-diphenyl-1-picrylhydrazyl (DPPH), general toxicity (Brine shrimp toxicity assay) , insecticidal ( Contact toxicity insecticidal assay) and antimalarial activities (hemebiocrystallization inhibition assay).

## Introduction

Natural chemical compounds presented as potent sources for new drug development. Among of the natural compounds, Iridoids and Iridoids like structure is a one of the significant phytochemical group structures that showed appropriate anti-parasitic potential in many investigations(1, 2). Similar structures are obvious in some important drugs such as Ivermectin as an important anti-parasitic drug. Parallel to this potential other activities such as radical scavenging and cytotoxic activity were detected in various Iridoids compounds(3-5).The Scrophulariaceae family consists of 220 genera known as the rich source of sugar esters and iridoid glycosides(6). The *Scrophularia *genus is represented by 60 species in the flora of Iran used as antipyretic, febrifuge and antibacterial, erythema, mouth dryness, constipation, prurigo, furunculosis, sore throat, ulcerous stomatitis, tonsillitis, and in the treatment of cancer (7). Previous investigations of *Scrophularia*species led to the isolation and characterization of Iridoid glucosides from *S.Buergeriana *(8, 9) and S. *ningpoensis*(10) and phenylpropanoid glycosides from S. *ningpoensis *(11). Based on these researches *Scrophularia* genus considered as a brilliant source of various iridoid compounds (12). Therefore, we designed this investigation for identification of *S. oxysepala *compounds especially acylated iridoids for antimalarial activity and others bioactivity. In this paper, we described the isolation and structure determination of chemical compounds and some biological activities of methanolic extract of the aerial part of *S. oxysepala.*

## Experimental

*General*

UV spectra were obtained in methanol in Shimadzu UV160-100. NMR spectra were recorded in CD_3_OD on a Bruker 200,400 MHz NMR spectrometer (200,400 MHz for^1^H and 200, 50 MHz for ^13^C) using residual solvent peak as the internal standard. Methanolic fractions were purified by a Knauer preparative HPLC coupled with UV-Visible–PDA detector (140-400 nm).

*Plant material*

The aerial parts of *S. oxysepala* were collected from East Azerbaijan province 30 kilometer to Kalibar town, Garehdagh mountain in the during flowering period. A voucher specimen (2821) has been deposited at the Herbarium of the Researches center for agriculture and natural resources, East Azerbaijan, Iran.

*Extraction and isolation*

**Figure 1:**
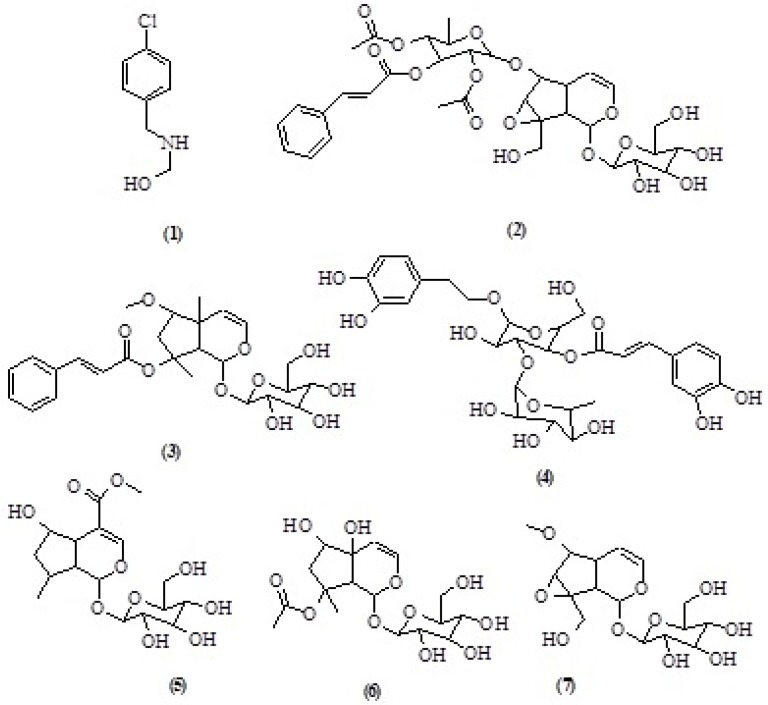
Structures of isolated compounds from *S.oxysepala*

The air-dried and powdered aerial parts of *S. oxysepala* (1800 g) were Soxhlet-extracted with n-hexane, dichloromethane (DCM), and methanol (MeOH) (2 L each). All these extracts were separately concentrated using a rotary evaporator at a maximum temperature of 45 °C.A portion of the MeOH extract (2 g) was subjected to solid phase extraction (SPE) on a Sep-Pak (10g) C_18_ cartridge using a step gradient of MeOH: water mixture (10:90, 20:80, 40:60, 60:40, 80:20, 100:0). The preparative HPLC (Dr. Mainsch GmbH ODS column 20 μM, 250 mm × 20 mm); liner gradient 0-45 min 20-90% methanol (MeOH) in water; isocratic gradient 90% MeOH in water during 45-50 min; liner gradient 50-52 min 90-100% MeOH in water; isocratic gradient 52-55 min 100 MeOH; liner gradient 55-58 min100-20% MeOH in water; isocratic gradient 20% MeOH in water during 58- 65 min; flow rate = 8 mL/min detection at 190- 400 nm to yielded: 2-(4-Chlorobenzyl amino) ethanol (8.2 mg, t_R_= 18.4 min) (1) from 10% MeOH SPE fraction, liner gradient 0-24 min 60-90% methanol in water; isocratic gradient 90% methanol in water during 20-40 min; liner gradient 40-43 min 90-100% MeOH in water; isocratic gradient 43-45 min 100% MeOH; liner gradient 45-47 min 100-60% MeOH; isocratic gradient 60% MeOH in water during 47-50 min; flow rate = 8 mL/min detection at 190-400 nm ) to yielded: Scrophuloside A (6.3mg , t_R_= 24.8 min)(13) (2), Harpagoside B (5.9 mg ,t_R_= 21.6 min)(14) (3) from 60% MeOH fraction, liner gradient 0-45 min30-80% methanol in water; isocratic gradient 80% methanol in water during 45-50 min; liner gradient 50-52 min 80-100% MeOH in water; isocratic gradient 52-58 min 100% MeOH; liner gradient 100-30% MeOH during 60-65 min; flow rate = 8 mL/min detection at 190- 400 nm to yielded Verbascoside (4.2 mg, t_R_= 19.1 min) (4), 5-hydroxyloganin (5.4 mg, t_R_=13.4 min) (5), 8-O-acetylharpagide (10.3 mg, t_R_= 21.2 min) (6) and 6-O-methyl,1-glucopyranosyl catalpol (13.6 mg, t_R_= 16.8 min) (7) from 40% MeOH fraction(Figure 1. Table 1, 2, 3).

*Hemebiocrystallization inhibition assay (Antimalarial activity)*

The potent antimalarial activity of methanolic extract and its fractions was evaluated by the method described by Fitch et.al. in 1999 with some modifications (15, 16). Briefly, different concentrations of methanolic extract and its fractions (0 to 3 mg/mL in 10% DMSO) were incubated with 300 μM of hematin (freshly dissolved in 0.1 M NaOH), 10 mM oleic acid and 10 μM HCl. The reaction volume was adjusted to 1 mL using 0.5 M sodium acetate buffer to obtain pH 5. Chloroquine diphosphate was used as a positive control. The samples were incubated overnight at 37 ^o^C with regular shaking. After incubation, samples were centrifuged (14,000 g, 10 min, at 21 °C) and the hemozoin pellet repeatedly washed with sonication (30 min, at 21 °C; FS100 bath sonicator; Decon Ultrasonics Ltd.) by 2.5% (w/v) SDS in phosphate buffered saline followed by a final wash in 0.1 M sodium bicarbonate (pH 9.0) until the supernatant was clear (Usually 3-5 washes). After the final wash, the supernatant was removed and the pellets were re-suspended in 1 mL 0.1 M NaOH. The hemozoin content was determined by measuring the absorbance at 400 nm (BeckmannDU640 spectrophotometer) using a 1 cm quartz cuvette. The results were recorded as % inhibition (I %) of heme polymerization/crystallization compared to positive control (chloroquine) using the following equation: I% = [(AB–AA)/AB] × 100, where AB: absorbance of blank; AA: absorbance of test samples.

*Free-radical-scavenging assay*

The electron donation ability of the extracts was determined spectrophotometrically by the bleaching of purple colored methanolic solution of DPPH (2, 2-diphenyl-1-picrylhydrazyl) as a reagent. 2, 2-Diphenyl-1-picrylhydrazyl (C_18_H_12_N_5_O_6_) was obtained from Sigma-Aldrich (Germany). DPPH (4 mg) was dissolved in MeOH (50 mL) to obtain the concentration of 80 μg/mL. Methanolic extract and its fractions were dissolved in MeOH to obtain a concentration of 1 mg/mL. Dilutions were made to obtain concentrations of 0.25, 0.125, 0.0625, 0.03125, 0.015625, 0.0078125, 0.00390625, 0.001953125, 0.000976563, 0.000488281 mg/mL. Diluted solutions were mixed with DPPH (1 mL) and allowed to stand for 30 min for any reaction to occur. The UV absorbance of samples and blank was recorded at 517 nm. The experiment was performed in triplicate and the average absorption was noted for each concentration. The same procedure was followed as the positive control, Quercetin. Percent inhibition of the free radical DPPH (I %) was calculated in the following equation:

I% = [(AB–AA)/AB] × 100, where AB: absorbance of blank; AA: absorbance of test samples. Concentration providing 50% inhibition (RC_50_) was calculated from the graph plotting inhibition percentage against test sample concentrations (17, 18).

*Contact toxicity insecticidal assay*

Adults of *Oryzeaphilus mercator* were collected from a laboratory culture. *O.mercator* was reared on a mixture of whole wheat and maize flour at the ratio 1:1 (0.5 Kg). All insects were reared at 27 ± 2 °C, 12% moisture content and continuous darkness for about 3 weeks without exposing to insecticides. Adults used in the experiments were 1-3 week old and of mixed sex. Methanolic extract and its fractions were dissolved in a suitable solvent to obtain a concentration of 1, 5, 10 and 15 mg/mL. The control was treated with pure solvents. After the solvent evaporation, 10 adults of *O.mercator* (Silvanidae) were placed in 20 mL glass vials maintained at 27 ± 2 °C, 12% moisture content and 12 h photo phase. The experimental design was completely randomized, with three replicates. Insect mortality was evaluated after 4, 8, 24, 48 h of exposure. Responses to treated vial versus control were converted to «percentage of mortality»(19).

*Brine shrimp toxicity assay*

Preliminary toxicity activity of MeOH extract and its fractions were determined by brine shrimp lethality assay according to Meyer *et al* method (20). Brine shrimp eggs (*Artemia salina*) were hatched in artificial seawater (3.8% sodium chloride solution) under artificial light at 28 °C with full aeration. After incubating for 24 h, the nauplii were attracted to one side of the beaker with a light source and collected with a Pasteur pipette. The MeOH extract and its fractions were initially dissolved in methanol. Solvent was evaporated and diluted with artificial seawater to provide the required concentrations (0.195, 0.39, 0.78, 1.562, 3.156, 6.25, 12.5, 25, 50 and 100 mg/mL). 15 nauplii were added to each tube containing the sample. After 24 h, the number of survivors was counted and recorded. The percentage of mortality in each tube and control was determined using the equation: % Mortality = (number of dead nauplii/ initial number of live nauplii ) × 100%. Lethality concentration fifties (LC_50_) for the assay were determined from the percentage mortality against logarithm of concentration graph.

## Results and discussion

The free-radical-scavenging activity of the MeOH extract and its fractions of the aerial parts of *S.oxysepala* was evaluated *in-vitro *by the DPPH assay. The hydrogen atoms or electrons donation ability of the extracts was determined spectrophotometrically by the bleaching of purple-colored methanolic solution of DPPH (2,2-diphenyl-1-picrylhydrazyl) as a reagent (17). The method is based on the reduction of methanolic DPPH solution in the presence of a hydrogen-donating scavenger through the formation of the non-radical form of DPPH (21).Tested extract and its fractions were capable to reduce the stable DPPH radical. A concentration-dependent activity pattern was observed (Table 4). Results of the brine shrimp cytotoxicity assay showed that 80% and 60% fractions showed mild cytotoxicity in comparison with podophyllotoxine as the standard (Table 4). These fractions have high content of acylated Iridoide glycosides. According to the previous research acylated Iridoide glycosides such as Scropolioside B and ScrospiosideA showed cytotoxicity (7).

**Table 1:** 1HNMR data of compounds 1-7.

	**1**	**2**	**3**	**4**	**5**	**6**	**7**
	δ H	δ H	δ H	δ H	δ H	δ H	δ H
1	_	4.89d(9.4)	6.18d(1.5)	_	5.67d(1.4)	5-5.2	6.08 brs
2	7.40*	_	_	6.70brs	_	-	-
3	7.40*	6.42dd(1.4 ,6)	6.44dd(1.5,6.4)	_	7.48s	6.38 dd (1.6, 6)	6.40 d (6.4)
4	_	5.04dd(6, 4.5)	_	_	_	5-5.2	5-5.2
5	7.40*	2.51m	_	6.70d(8)	_	2.34	-
6	7.40*	4.09d(8)	_	6.59dd(1.6,8)	3.94t(4.5)	-	3.9
7	3.94s	3.79brs	2.32d(15.2)	2.83t(7.2)	2.64,2.09dd(5.6,14)	3.78 brs	1.26-2.21
8	_	_	_	3.8-4.1*	2.25m	-	-
9	_	2.60dd(9.4,8)	2.97d(1)	_	2.82*	2.59 dd (1.6, 7.6)	2.86 brs
10	_	4.20, 3.85d(13)	1.5s	_	0.98d(7.2)	3.85, 4.19 dd (13.2, 13.2)	1.46 s
11	-	-	-	-	-	3.49 s	-
12	-	-	-	-	-	-	2.02 s
1'	2.89t(5.4)	4.83d(7.8)	4.65d(7.9)	4.40d(8)	4.59d(7.9)	4.81 d (7.6)	4.61 d(7.8)
2'	3.75t(5.4)	_	_	3.2-3.8*	3.2-3.8*	3.1-3.4	3.1-3.8
3'	_	3.46d(8.6)	_	3.2-3.8*	3.2-3.8*	3.1-3.4	3.1-3.8
4'	_	3.42	_	4.1-4.8**	3.2-3.8*	3.1-3.4	3.1-3.8
5'	_	3.53m	_	3.2-3.8*	3.2-3.8*	3.1-3.4	3.1-3.8
6'	_	_	_	3.2-3.8*	3.94,3.70dd(10,1.5)	3.68, 3.94 dd (12, 12)	3.1-3.8
1''	_	4.97d(1.5)	_	_	_	-	-
2''	_	5.31	_	7.06d(1.8)	_	-	-
3''	_	5.39 d(9.6)	_	_	_	-	-
4''	_	5.16dd(10, 9.6)	_	_	_	-	-
5''	_	_	_	6.84d(8)	_	-	-
6''	_	1.21d(6)	_	6.98dd(1.8,8)	_	-	-
7''	_	_	7.72d(16)	7.64d(16)	_	-	-
8''	_	2.10s	6.56d(16)	6.32d(16)	_	-	-
9''	_	_	_	_	_	-	-
10''	_	1.99s	_	_	_	-	-
1'''	_	_	_	5.19d(1.5)	_	-	-
2'''	_	7.48*m	_	3.2-3.8*	_	-	-
3'''	_	7.34*m	_	3.2-3.8*	_	-	-
4'''	_	7.34*m	_	3.2-3.8*	_	-	-
5'''	_	7.34*m	_	3.2-3.8*	_	-	-
6'''	_	7.48*m	_	1.11d(6)	_	-	-
7'''	_	7.69d(16)	_	_	_	-	-
8'''	_	6.48d(16)	_	_	_	-	-
9'''	_	_	_	_	_	-	-
O-CH3	_	_	3.369s	_	3.73s	-	-

**Table 2:** ^13^CNMR data of compounds 1-7.

	**1**	**2**	**3**	**4**	**5**	**6**	**7**
	δC	δC	δC	δC	δC	δC	δC
1	135.0	93.72	93.21	130.00	94.11	93.94	91.07
2	128.42	_	_	113.76	_	-	-
3	130.43	141.09	142.56	144.71	153.18	140.65	142.45
4	133.53	101.76	105.44	143.26	_	102.62	105.45
5	130.43	35.73	_	115.68	46.29	35.93	73.11
6	128.42	83.42	76.20	119.83	70.29	87.03	76.25
7	49.65	57.99	44.83	35.15	42.09	56.87	44.60
8	_	65.14	87.39	70.92	88.4	65.35	87.22
9	_	41.85	54.13	_	48.24	41.70	54.03
10	_	59.98	21.24	_	17.41	61.27	20.79
11	51.21	98.29	98.59	101.63	98.24	58.05	-
12	58.72	73.41	73.13	74.78	72.95	-	21.07
1'	_	77.23	76.20	80.25	76.43	98.27	98.49
2'	_	70.85	72.05	69.11	70.20	73.28	71.91
3'	_	76.25	76.75	74.60	77.04	76.97	76.25
4'	_	61.57	61.51	60.93	61.41	70.13	70.25
5'	_	96.25	134.35	126.20	_	76.09	76.76
6'	_	69.35	127.81	113.76	_	60.02	61.40
1''	_	70.37	128.62	145.42	_	-	-
2''	_	68.89	130.12	148.39	_	-	-
3''	_	66.63	128.62	115.68	_	-	-
4''	_	16.30	127.81	121.82	_	-	-
							
							
	**1**	**2**	**3**	**4**	**5**	**6**	**7**
5''	_	170.21	144.72	146.61	_	-	-
6''	_	19.33	118.69	115.08	_	-	-
7''	_	170.43	167.34	166.87	_	-	-
8''	_	19.33	_	_	_	-	-
9''	_	130.41	_	102.77	_	-	-
10''	_	128.00	_	70.92	_	-	-
1'''	_	128.67	_	70.60	_	-	-
2'''	_	134.07	_	72.35	_	-	-
3'''	_	128.67	_	69.11	_	-	-
4'''	_	128.00	_	17.05	_	-	-
5'''	_	145.97	_	_	_	-	-
6'''	_	116.48	_	_	_	-	-
7'''	_	165.82	_	_	_	-	-
8'''	_	_	49.79	_	50.09	-	-
9'''						-	-
O-CH3						-	-

**Table 3:** HMBC data of compound 2

	^1^ *H-NMR*	^13^ *C-NMR*	*HMBC*	
			^2^J_CH_	^3^J_CH_
	δ H	δC		
1	4.89d(9.4)	93.72	C-9	C-1ʹ, C-8
2	_	_	_	_
3	6.42dd(1.4 ,6)	141.09	_	_
4	5.04dd(6, 4.5)	101.76	C-3	_
5	2.51m	35.73	_	_
6	4.09d(8)	83.42	C-5 , C-7	C-8 , C-1ʹ
7	3.65brs	57.99	C-6	C-5
8	_	65.14	_	_
9	2.60dd(9.4,8)	41.85	_	_
10	4.20, 3.85d(13)	59.98	_	_
1'	4.83d(7.8)	98.29	_	_
2'	_	73.41	_	_
3'	3.46d(8.6)	77.23	_	_
4'	3.42	70.85	_	_
5'	3.53m	76.25	_	_
6'	_	61.57	_	_
1''	4.97d(1.5)	96.25	C-2 ʹʹ	C-3 ʹʹ , C-5 ʹʹ , C-6
2''	5.31	69.35	C-3 ʹʹ	C-7 ʹʹ , C-4 ʹʹ
3''	5.39 d(9.6)	70.37	C-2 ʹʹ, C-4 ʹʹ	C-9ʹʹʹ
4''	5.16dd(10, 9.6)	68.89	C-3 ʹʹ , C-5 ʹʹ	C-9 ʹʹ
	^1^ *H-NMR*	^13^ *C-NMR*	*HMBC*	
5''	_	66.63	_	_
6''	1.21d(6)	16.30	_	_
7''	_	170.21	_	_
8''	2.10s	19.33	C-7 ʹʹʹ	_
9''	_	170.43	_	_
10''	1.99s	19.33	C-9 ʹʹʹ	_
1'''	_	130.41	_	_
2'''	7.48*m	128.00	C-3 ʹʹʹ	C-7 ʹʹʹ, C-4 ʹʹʹ
3'''	7.34*m	128.67	C-4 ʹʹʹ, C-2 ʹʹʹ	_
4'''	7.34*m	134.07	C-5ʹʹʹ	C-2 ʹʹʹ
5'''	7.34*m	128.67	C-4 ʹʹʹ, C-6 ʹʹʹ	_
6'''	7.48*m	128.00	C-5ʹʹʹ	C-7 ʹʹʹ
7'''	7.69d(16)	145.97	C-9 ʹʹʹ, C-8 ʹʹʹ	C-2 ʹʹʹ
8'''	6.48d(16)	116.48	C-9 ʹʹʹ	C-1 ʹʹʹ
9'''	_	165.82	_	_

**Table 4:** Free-radical-scavenging and cytotoxic activity of methanolic extract and its fractions of *S .oxysepala*

***Sample***	***Brine shrimp toxicity***	***Antioxidant activity***
	LC_50_(µg/mL)	RC_50_(mg/mL)
MeOH extract	641.14	0.957
10% Fraction	835.68	0.247
20% Fraction	768.20	0.137
40% Fraction	503.32	0.0247
60% Fraction	201.04	0.0438
80% Fraction	419.36	0.640
100% Fraction	512.61	0.825
Standard	Podophyllotoxine 2.58	Quercetin 0.0249

**Table 5:** Contact toxicity assay of the fractions and MeOH extract of *S.oxysepala*

**Hours**	**Conc.(mg/mL)**	**MeOH extract**	**40 % fraction**	**60 % fraction**
		**% Mortality**	**% Mortality**	**% Mortality**
4	1	0	0	0
	5	3.3	0	0
	10	0	3.3	0
	15	3.3	3.3	10
8	1	3.3	3.3	3.3
	5	0	3.3	10
	10	3.3	6.6	10
	15	3.3	10	20
24	1	6.6	3.3	10
	5	3.3	6.6	10
	10	6.6	10	20
	15	3.3	10	20
48	1	6.6	3.3	10
	5	10	10	20
	10	10	10	30
	15	6.6	10	30

Malaria-infected erythrocytes are characterized by a high rate of production of ferriprotoporphyrin IX (heme) as a result of the ingestion and digestion of host cell hemoglobin (22). Parasite utilizes hemoglobin as food source during intra erythrocytes development and proliferation (23). Released heme is a toxic compound (24). For the detoxification of heme, crystals of hemozoin in the food vacuoles of malaria parasites are formed that commonly known as malaria pigment (25-27). Chloroquine and most of other antimalarial compounds inhibit β-hematin formation under different conditions (26). The hemebiocrystallization inhibition assay is based on inhibition of β-hematin formation (28, 29).In this study the MeOH extract and its fractions of *S. oxysepala* were assessed for potential antimalarial activity by the hemebiocrystallization inhibition assay. The 80% and 60% fractions showed significant antimalarial effects compared to other fractions and blank. At 1-2 mg/mL concentrations heme biocrystallization inhibition was higher than the blank. The IC_50_ values of 80% and 60% fractions were 0.936 and 0.754 mg/mL while the IC_50_ of positive control (chloroquine) was 0.052 mg/mL. This is the first report on the insecticidal property of the *S. oxysepala* that were evaluated by contact toxicity assay. The results showed that 40% and 60% fractions had potent insecticidal activity compared to other fractions (Table 5). 
